# Supplementary material for: Progerin accelerates atherosclerosis by inducing endoplasmic reticulum stress in vascular smooth muscle cells
Source: EMBO Mol Med. 2019 Mar 12;11(4):e9736. doi: 10.15252/emmm.201809736 (PMC6460349; doi:10.15252/emmm.201809736)
Supplement: Supplementary file 1 — Appendix [file EMMM-11-e9736-s001.pdf]

APPENDIX

Table of contents:

- **Appendix Figure S1**. Genes within endoplasmic reticulum stress and the unfolded protein response pathway differentially expressed in medial aorta from progeria mouse models.

- **Appendix Figure S2**. Prolonged treatment of *Apoe*$^{-/-}$*Lmna*$^{+/+}$ mice with tauroursodeoxycholic acid (TUDCA) does not significantly affect atherosclerosis and has only minor effect on the body weight.

- **Appendix Figure S3**. Progerin expression in the aorta of *Apoe*$^{-/-}$*Lmna*$^{LCS/LCS}$*SM22αCre* mice during embryonic development.

- **Appendix Figure S4**. Progerin expression in the intestine of *Apoe*$^{-/-}$*Lmna*$^{LCS/LCS}$*SM22αCre* mice during embryonic development.

- **Appendix Figure S5**. Progerin expression in the kidney of *Apoe*$^{-/-}$*Lmna*$^{LCS/LCS}$*SM22αCre* mice during embryonic development.

- **Appendix Figure S6**. Progerin expression in the heart of *Apoe*$^{-/-}$*Lmna*$^{LCS/LCS}$*SM22αCre* mice during embryonic development.

- **Appendix Figure S7**. Kidneys of 26-week-old *Apoe*$^{-/-}$*Lmna*$^{LCS/LCS}$*SM22αCre* mice have very low expression of progerin, predominantly limited to blood vessels.

- **Appendix Figure S8**. Progerin expression in the muscularis externa in the intestine of 12- and 26-week-old *Apoe*$^{-/-}$*Lmna*$^{LCS/LCS}$*SM22αCre* mice does not cause smooth muscle cell loss.

- **Appendix Table S1.** The exact *P* values for Figure 2.

- **Appendix Table S2**. Primer sequence for quantitative real-time PCR (mouse genes).

- **Appendix Table S3**. Primer sequence for quantitative real-time PCR (human genes).

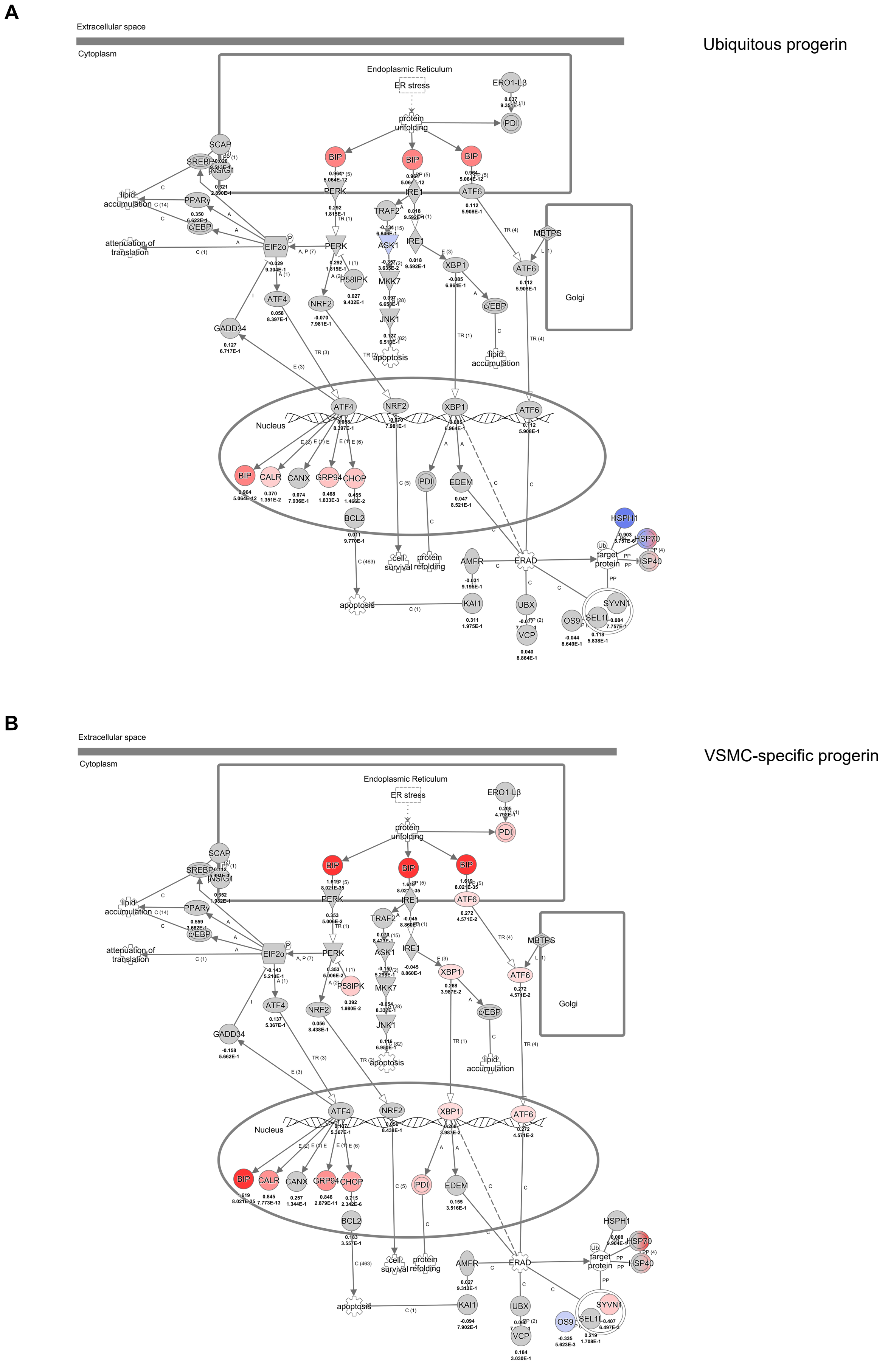

Appendix Figure S1. Genes within endoplasmic reticulum stress and the unfolded protein response pathway differentially expressed in medial aorta from progeria mouse models. (A) Ubiquitous progerin expression (*Apoe*$^{-/-}$*Lmna*$^{G609G/G609G}$). **(B)** Vascular smooth muscle cell-specific progerin expression (*Apoe*$^{-/-}$*Lmna*$^{LCS/LCS}$*SM22αCre*). Red – upregulated; blue – downregulated. Color intensity corresponds to fold change level. Below each gene, numbers describe changes in expression (log$_2$[fold-change] and corresponding adjusted *P*-value).

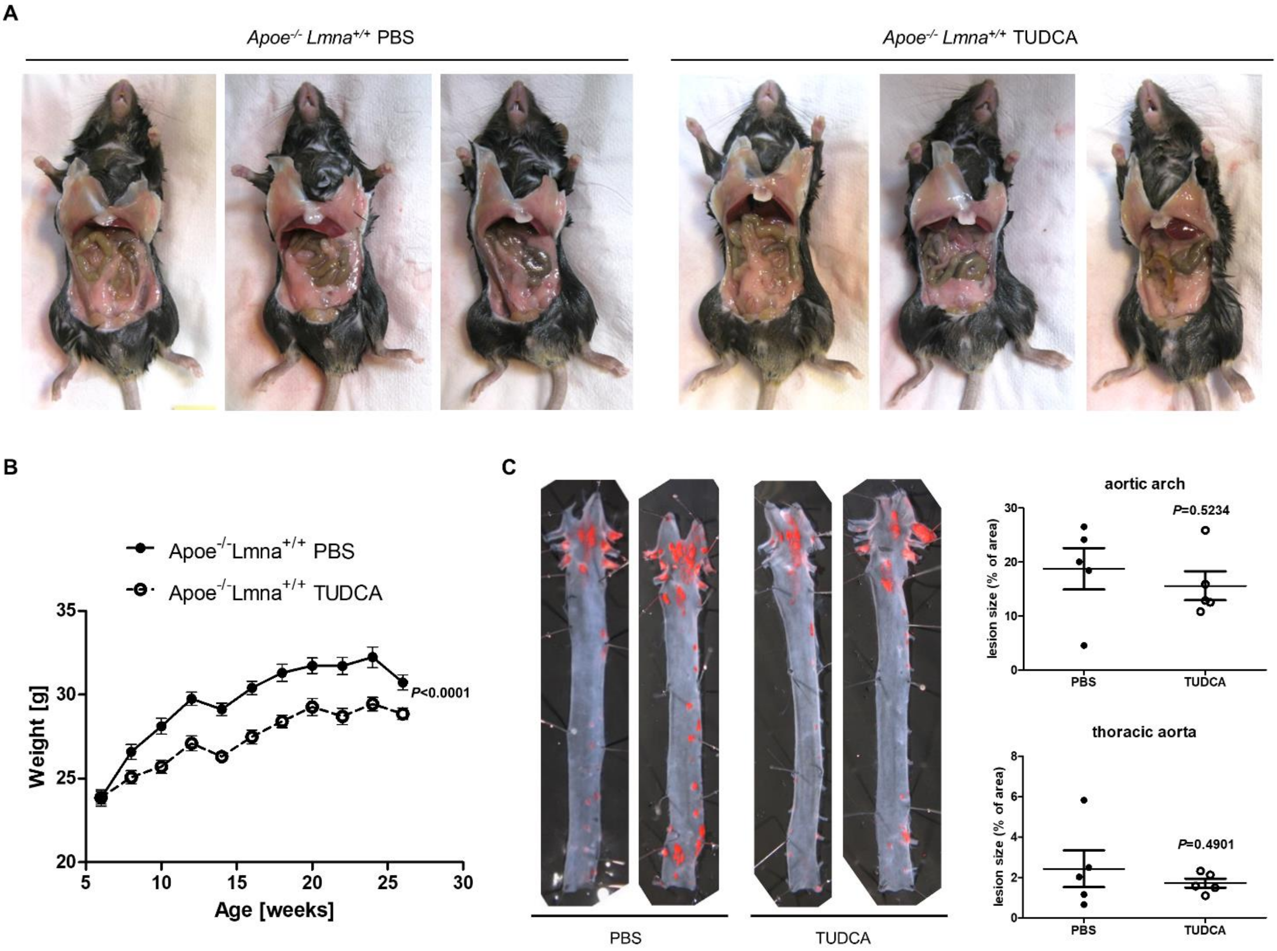

Appendix Figure S2. Prolonged treatment of *Apoe*$^{-/-}$*Lmna*$^{+/+}$ mice with tauroursodeoxycholic acid (TUDCA) does not significantly affect atherosclerosis and has only minor effect on the body weight. *Apoe*$^{-/-}$*Lmna*$^{+/+}$ mice fed normal chow received either TUDCA (400 mg/kg) or PBS intraperitoneal injections 3 times a week starting at 6 weeks of age. Mice were sacrificed at 27 weeks of age. **(A)** Representative photographs of intraperitoneal cavity of TUDCA-treated and untreated mice. **(B)** Body weight curves for TUDCA-treated and untreated mice. **(C)** Representative images of Oil Red O-stained aortas of TUDCA-treated and untreated mice; graphs show quantification of atherosclerosis burden in aortic arch and thoracic aorta. n=5. Data are mean ± SEM. Statistical differences were analyzed by two-tailed paired *t*-test in **B** and two-tailed unpaired *t*-test with Welch's correction in **C**.

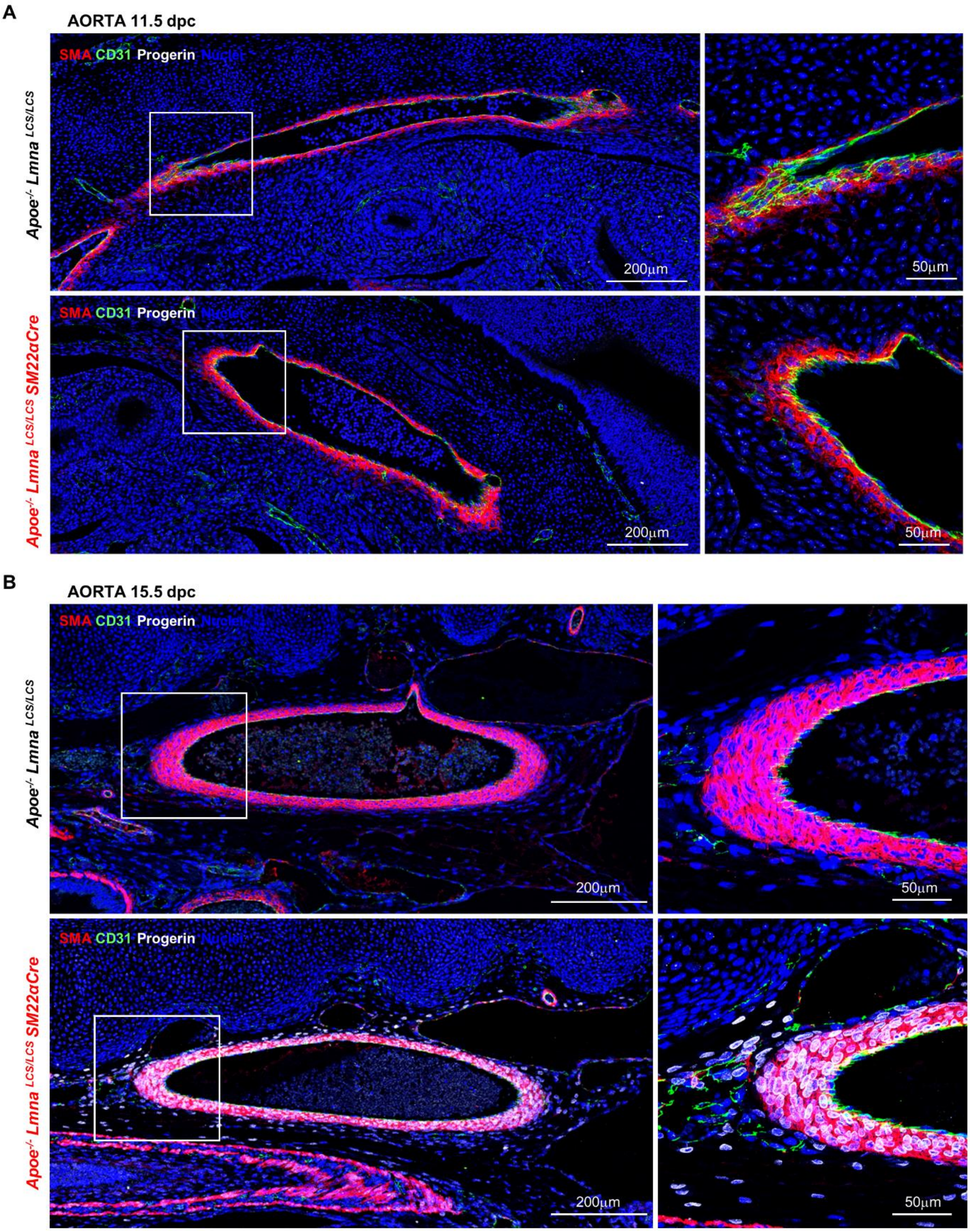

Appendix Figure S3. Progerin expression in the aorta of *Apoe$^{-/-}$Lmna$^{LCS/LCS}$SM22αCre* mice during embryonic development. Embryos were collected at 11.5 and 15.5 days post coitum (dpc), embedded in paraffin, sectioned longitudinally and stained with Hoechst 33342 nucleic acid stain (visualized in blue), anti-smooth muscle α-actin (SMA, visualized in red) and anti-progerin (visualized in white) antibodies. Confocal microscopy images show aorta at 11.5 **(A)** and 15.5 dpc **(B)**.

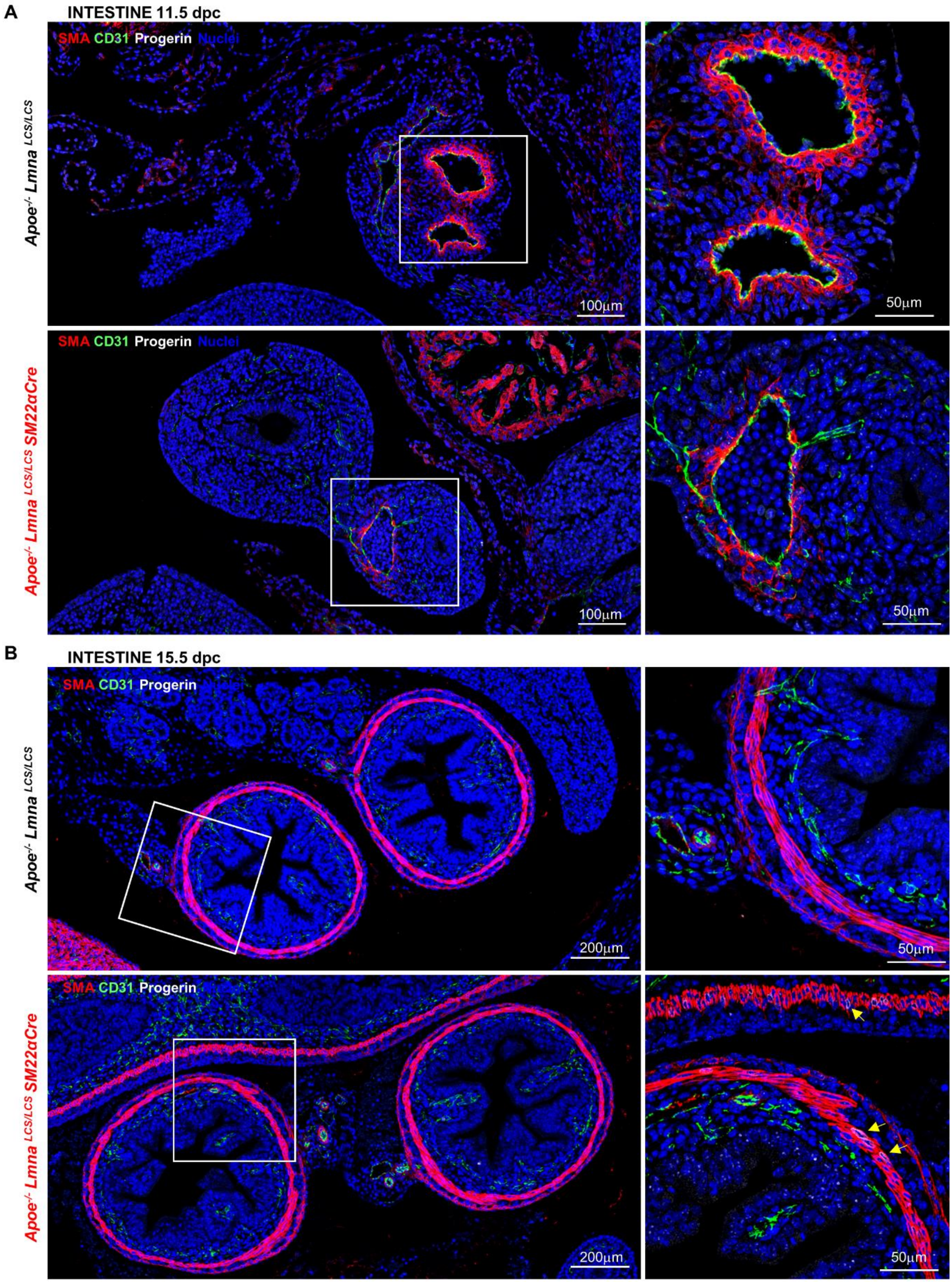

Appendix Figure S4. Progerin expression in the intestine of *Apoe*$^{-/-}$*Lmna*$^{LCS/LCS}$*SM22αCre* mice during embryonic development. Embryos were collected at 11.5 and 15.5 days post coitum (dpc), embedded in paraffin, sectioned longitudinally and stained with Hoechst 33342 nucleic acid stain (visualized in blue), anti-smooth muscle α-actin (SMA, visualized in red) and anti-progerin (visualized in white) antibodies. Confocal microscopy images show intestine at 11.5 **(A)** and 15.5 dpc **(B)**. Yellow arrows indicate examples of progerin-positive nuclei.

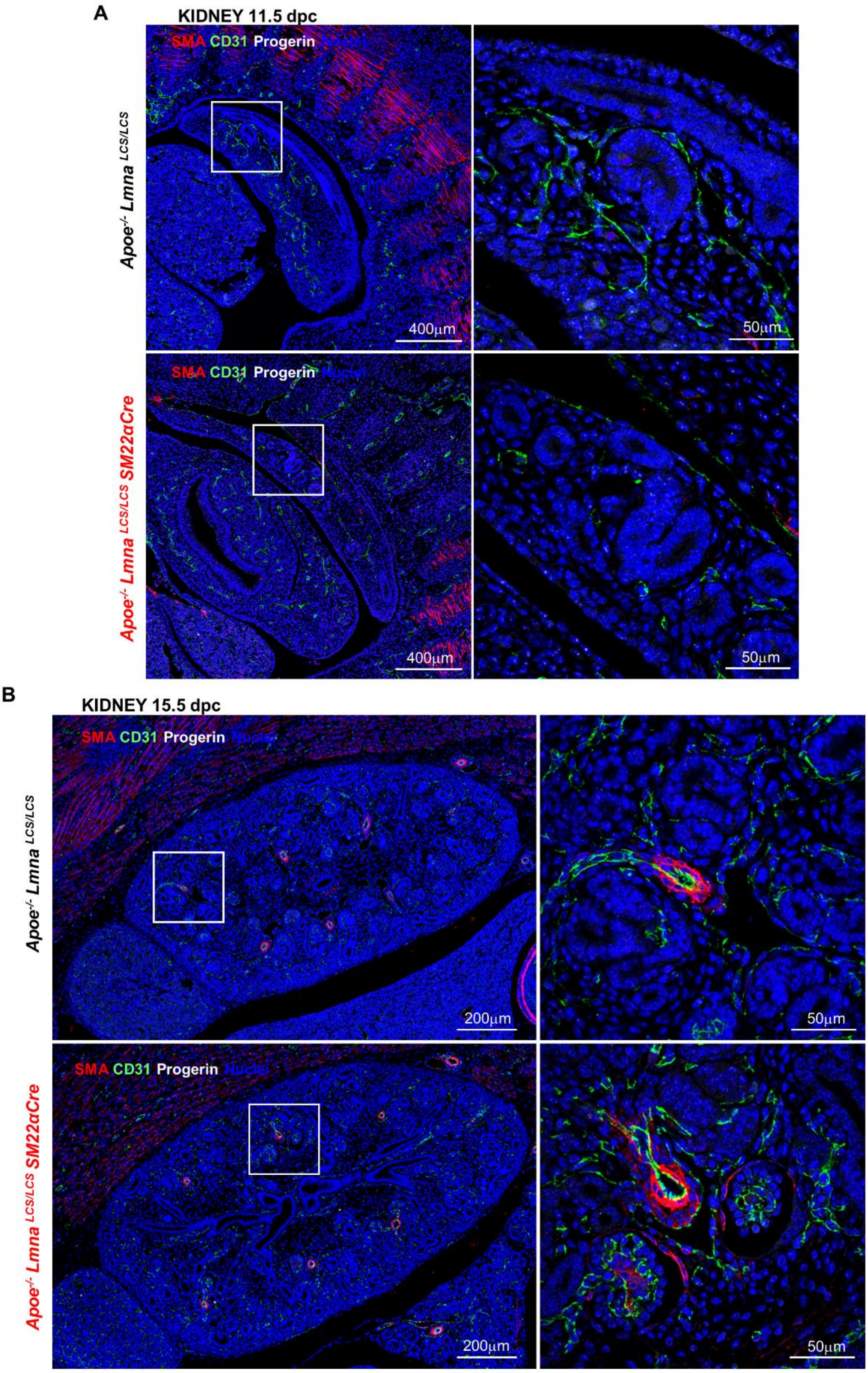

Appendix Figure S5. Progerin expression in the kidney of *Apoe$^{-/-}$Lmna$^{LCS/LCS}$SM22αCre* mice during embryonic development. Embryos were collected at 11.5 and 15.5 days post coitum (dpc), embedded in paraffin, sectioned longitudinally and stained with Hoechst 33342 nucleic acid stain (visualized in blue), anti-smooth muscle α-actin (SMA, visualized in red) and anti-progerin (visualized in white) antibodies. Confocal microscopy images show kidney at 11.5 **(A)** and 15.5 dpc **(B)**.

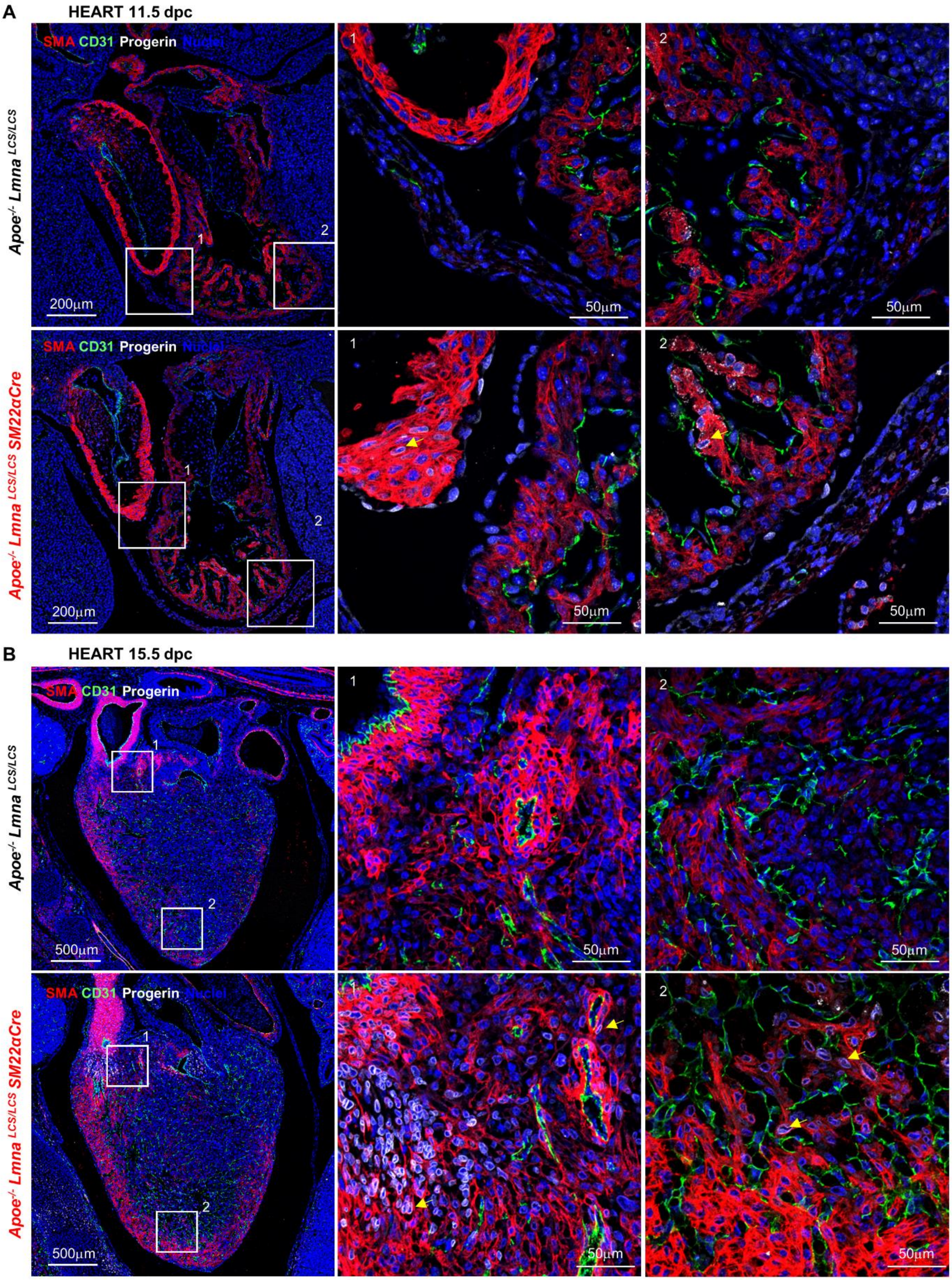

Appendix Figure S6. Progerin expression in the heart of ***Apoe$^{-/-}$Lmna$^{LCS/LCS}$SM22αCre*** **mice during embryonic development.** Embryos were collected at 11.5 and 15.5 days post coitum (dpc), embedded in paraffin, sectioned longitudinally and stained with Hoechst 33342 nucleic acid stain (visualized in blue), anti-smooth muscle α-actin (SMA, visualized in red) and anti-progerin (visualized in white) antibodies. Confocal microscopy images show heart at 11.5 **(A)** and 15.5 dpc **(B)**. Yellow arrows indicate examples of progerin-positive nuclei.

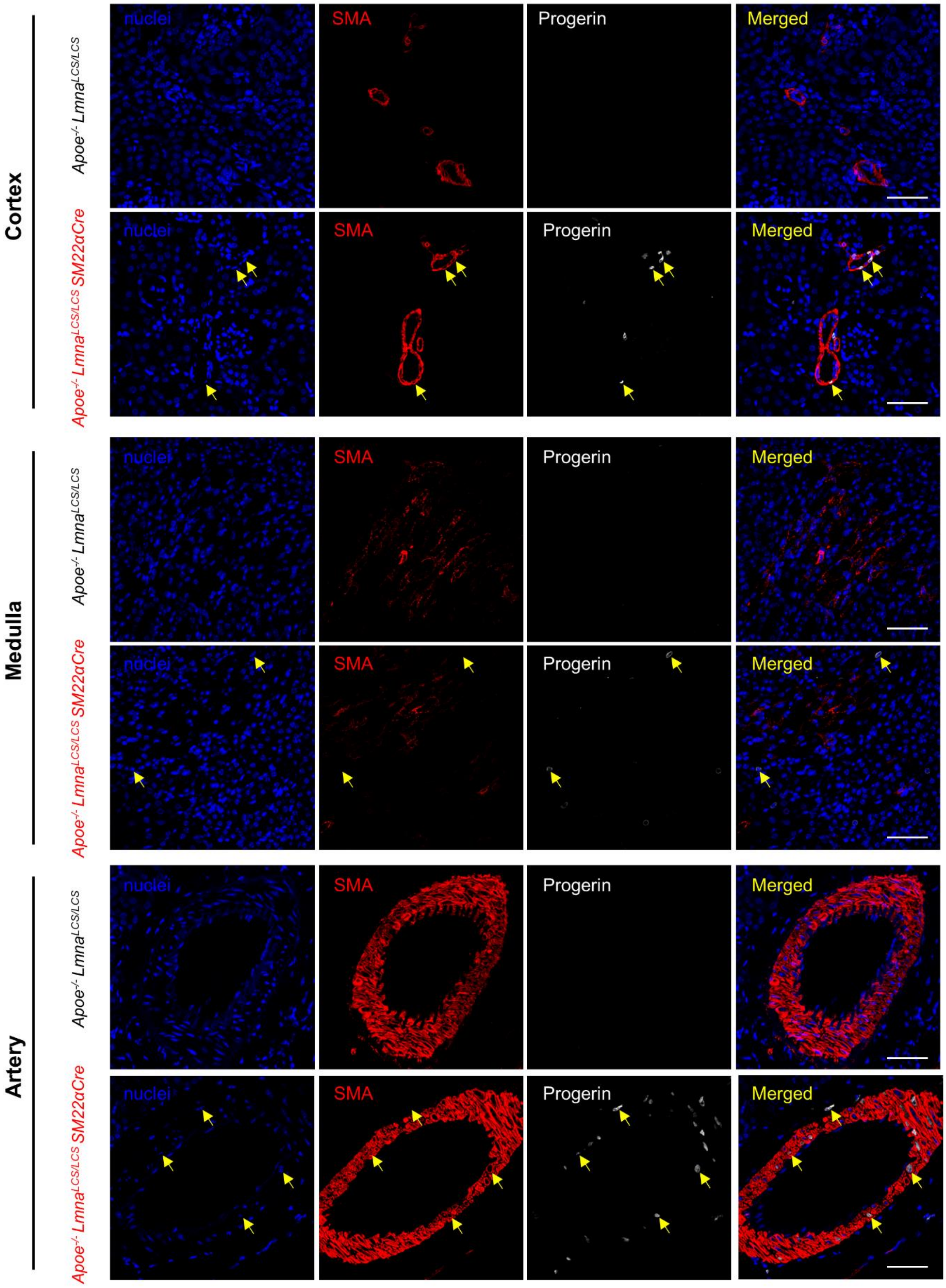

Appendix Figure S7. Kidneys of 26-week-old *Apoe*$^{-/-}$*Lmna*$^{LCS/LCS}$*SM22αCre* mice have very low expression of progerin, predominantly limited to blood vessels. Male mice were fed normal chow and sacrificed at 26 weeks of age. Kidneys were embedded in paraffin and sectioned for immunofluorescence studies. Images show representative sections of the kidney (cortex, medulla and artery) stained against smooth muscle α-actin (SMA, visualized in red) and progerin (visualized in white). Nuclei were stained with Hoechst 33342 (visualized in blue). 4-6 animals per genotype were analyzed. Scale bar 50 µm. Yellow arrows indicate examples of progerin-positive nuclei.

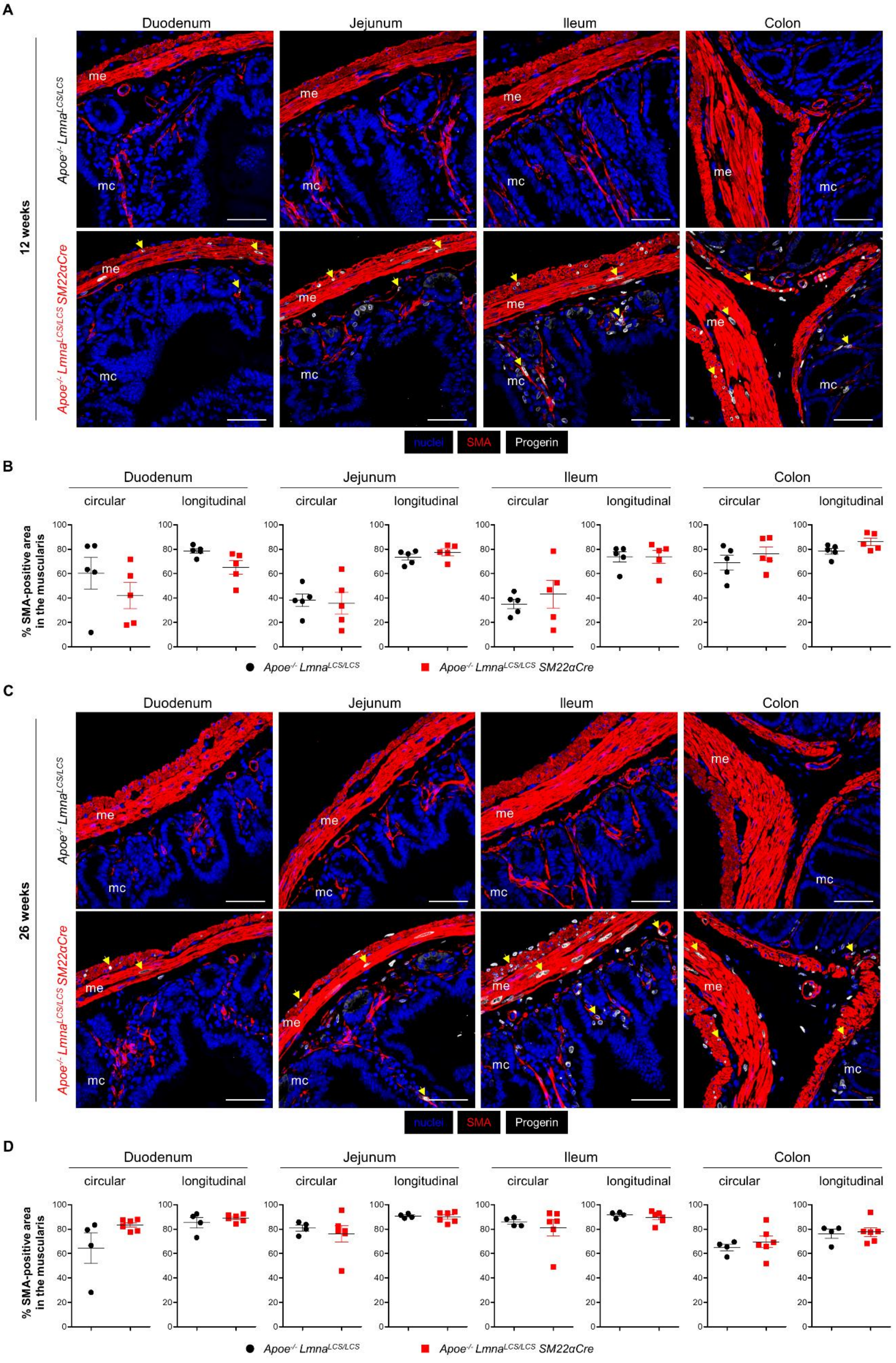

Appendix Figure S8. Progerin expression in the muscularis externa in the intestine of 12- and 26-week-old *Apoe*$^{-/-}$ *Lmna*$^{LCS/LCS}$*SM22αCre* mice does not cause smooth muscle cell loss. Male mice were fed normal chow and sacrificed at 12 or 26 weeks of age. Various regions of the intestine (duodenum, jejunum, ileum, and colon) were embedded in the paraffin and sectioned for immunofluorescence studies. **(A, C)** Images show transversal sections of the intestine stained against smooth muscle α-actin (SMA, visualized in red) and progerin (visualized in white) in 12- **(A)** and 26-week-old **(C)** animals. Nuclei were stained with Hoechst 33342 (visualized in blue). Scale bar 50 µm. me – muscularis externa; mc – mucosa. Yellow arrows indicate examples of progerin-positive nuclei. **(B, D)** Graphs represent quantification of the percentage of SMA-positive area in the muscularis externa in both circular and longitudinal muscle of 12- **(B)** and 26-week-old **(D)** animals. n=4-6. Data are presented as mean ± SEM. Statistical differences were analyzed by two-tailed *t*-test.

Appendix Table S1. The exact *P* values for Figure 2.

***Apoe*$^{-/-}$ *Lmna*$^{+/+}$ mice vs *Apoe*$^{-/-}$ *Lmna*$^{G609G/G609G}$ mice**

	Fig 2B	Fig 2E			
**Gene**	**medial aorta**	**kidney**	**heart**	**spleen**	**liver**
Calr	0.0308	0.0107	0.3802	0.3078	0.0398
Hspa5	0.0036	0.0885	0.1378	0.1533	0.9737
Hsp90b1	0.2547	0.0038	0.0478	0.0313	0.988
Pdia4	0.0155	0.0005	0.061	0.0789	0.535
Dnajb9	0.0047	0.009	0.3871	0.0218	0.1289
Ddit3	0.0268	0.127	0.0062	0.692	0.9054
	Unpaired one-tailed t-test	Unpaired two-tailed t-test			

***Apoe*$^{-/-}$ *Lmna*$^{LCS/LCS}$ mice vs *Apoe*$^{-/-}$ *Lmna*$^{LCS/LCS}$ *SM22αCre* mice**

	Fig 2C	Fig 2F			
**Gene**	**medial aorta**	**kidney**	**heart**	**spleen**	**liver**
Calr	0.0033	0.0893	0.4297	0.7058	0.5468
Hspa5	0.0001	0.5392	0.7634	0.4838	0.6171
Hsp90b1	0.0125	0.2348	0.813	0.9825	0.4096
Pdia4	0.0002	0.0248	0.8589	0.5831	0.3761
Dnajb9	0.003	0.2825	0.333	0.4982	0.4064
Ddit3	0.0091	0.9852	0.7131	0.4952	0.1091
	Unpaired one-tailed t-test	Unpaired two-tailed t-test			

control cells vs HGPS patient-derived cells

	Fig 2D
**Gene**	**fibroblasts**
DDIT3	0.0258
HSP90B1	0.0591
HSPA5	0.0525
DNAJC3	0.06035
ATF4	0.0053
EIF2AK3	0.0277
ERN1	0.0476
CALR	0.0066
ERO1B	0.00255
PPP1R15A	0.05565
Spliced/Total XBP1	0.01845
PBGD	0.0633
	One-sample one-tailed t-test

Appendix Table S2. Primer sequence for quantitative real-time PCR (mouse genes).

Gene	Forward primer	Reverse primer
Calr	5′-CCAGAAATTGACAACCCTGAA-3′	5′-CCTTAAGCCTCTGCTCCTCAT-3′
Ddit3	5′-ATATCTCATCCCCAGGAAACG-3′	5′-CTCCTGCTCCTTCTCCTTCAT-3′
Dnajb9	5′-AGAATTAATCCTGGCCTCCAA-3′	5′-GGCATCCGAGAGTGTTTCATA-3′
Hspa5	5′-GTGGGAGGAGTCATGACAAAA-3′	5′-TTCAGCTGTCACTCGGAGAAT-3′
Hsp90b1	5′-AGTGGAAGAGGACCTGGGTAA-3′	5′-AGCGAGTGCATTTTCATCAGT-3′
Pdia4	5′-TCCTGAAGGATGGAGATGATG-3′	5′-ACCTGGGCTCATACTTGGACT-3′
Gusb	5′-GAGTATGGAGCAGACGCAATC-3′	5′-TCCGACCACGTATTCTTTACG-3′
Hprt	5′-AGGCCAGACTTTGTTGGATTT-3′	5′-GGCTTTGTATTTGGCTTTTCC-3′

Appendix Table S3. Primer sequence for quantitative real-time PCR (human genes).

Gene	Forward primer	Reverse primer
DDIT3	5’-GGAGCTGGAAGCCTGGTATGAGG-3’	5’-TCCCTGGTCAGGCGCTCGATTTCC-3’
HSP90B1	5’-CTCACCATTTGGATCCTGTGTG-3’	5’-CACATGACAAGATTTTACATCAAGA-3’
HSPA5	5′-GCCGAGGAGGAGGACAAGAA-3′	5′-CACAGACGGGTCATTCCACG-3′
DNAJC3	5’-GAGGTTTGTGTTTGGGATGCAG-3’	5’-GCTCTTCAGCTGACTCAATCAG-3’
ATF4	5’-GCTAAGGCGGGCTCCTCCGA-3’	5’-ACCCAACAGGGCATCCAAGTCG-3’
EIF2AK3	5’-AATGCCTGGGACGTGGTGGC-3’	5’-TGGTGGTGCTTCGAGCCAGG-3’
ERN1	5’-TGCTTAAGGACATGGCTACCATCA-3’	5’-CTGGAACTGCTGGTGCTGGA-3’
CALR	5’-GCCAAGGACGAGCTGTAGAGAG-3’	5’-GGTGAGGGCTGAAGGAGAATC-3’
ERO1B	5’-TTCTGGATGATTGCTTGTGTGAT-3’	5 -GGTCGCTTCAGATTAACCTTGT-3’
PPP1R15A	5 -CCCAGAAACCCCTACTCATGATC-3’	5’-GCCCAGACAGCCAGGAAAT-3’
Spliced XBP1	5 -CTGAGTCCGAATCAGGTGCAG-3’	5’-ATCCATGGGGAGATGTTCTGG-3’
Total XBP1	5’-CAGCGCTTGGGGATGGATGC-3’	5’-CCATGGGGAGATGTTCTGGA -3’
GAPDH	5’-CTGTTGCTGTAGCCAAATTCGT-3’	5’-ACCCACTCCTCCACCTTTGA-3’
