# Supplementary material for: Progerin accelerates atherosclerosis by inducing endoplasmic reticulum stress in vascular smooth muscle cells
Source: EMBO Mol Med. 2019 Mar 12;11(4):e9736. doi: 10.15252/emmm.201809736 (PMC6460349; doi:10.15252/emmm.201809736)
Supplement: Supplementary file 2 — Expanded View Figures PDF [file EMMM-11-e9736-s002.pdf]

Expanded View Figures

Figure EV1. Pathways affected by lack of lamin A do not overlap with those induced by progerin expression.

A–C Stacked bar charts representing pathways significantly changed after applying the Benjamini–Hochberg correction for multiple testing in three comparisons: (A) $Apoe^{-/-}Lmna^{G609G/G609G}$ (ubiquitous progerin) versus $Apoe^{-/-}Lmna^{+/+}$ (both lamin A and lamin C), (B) $Apoe^{-/-}Lmna^{LCS/LCS}SM22\alpha Cre$ (vascular smooth muscle cell (VSMC)-specific progerin) versus $Apoe^{-/-}Lmna^{LCS/LCS}$ (lamin C only, no lamin A), and (C) $Apoe^{-/-}Lmna^{LCS/LCS}$ (lamin C only, no lamin A) versus $Apoe^{-/-}Lmna^{+/+}$ (both lamin A and lamin C). The numbers of genes in each category (from the Ingenuity Pathway Analysis data base) are indicated above the bars.

© 2019 The Authors

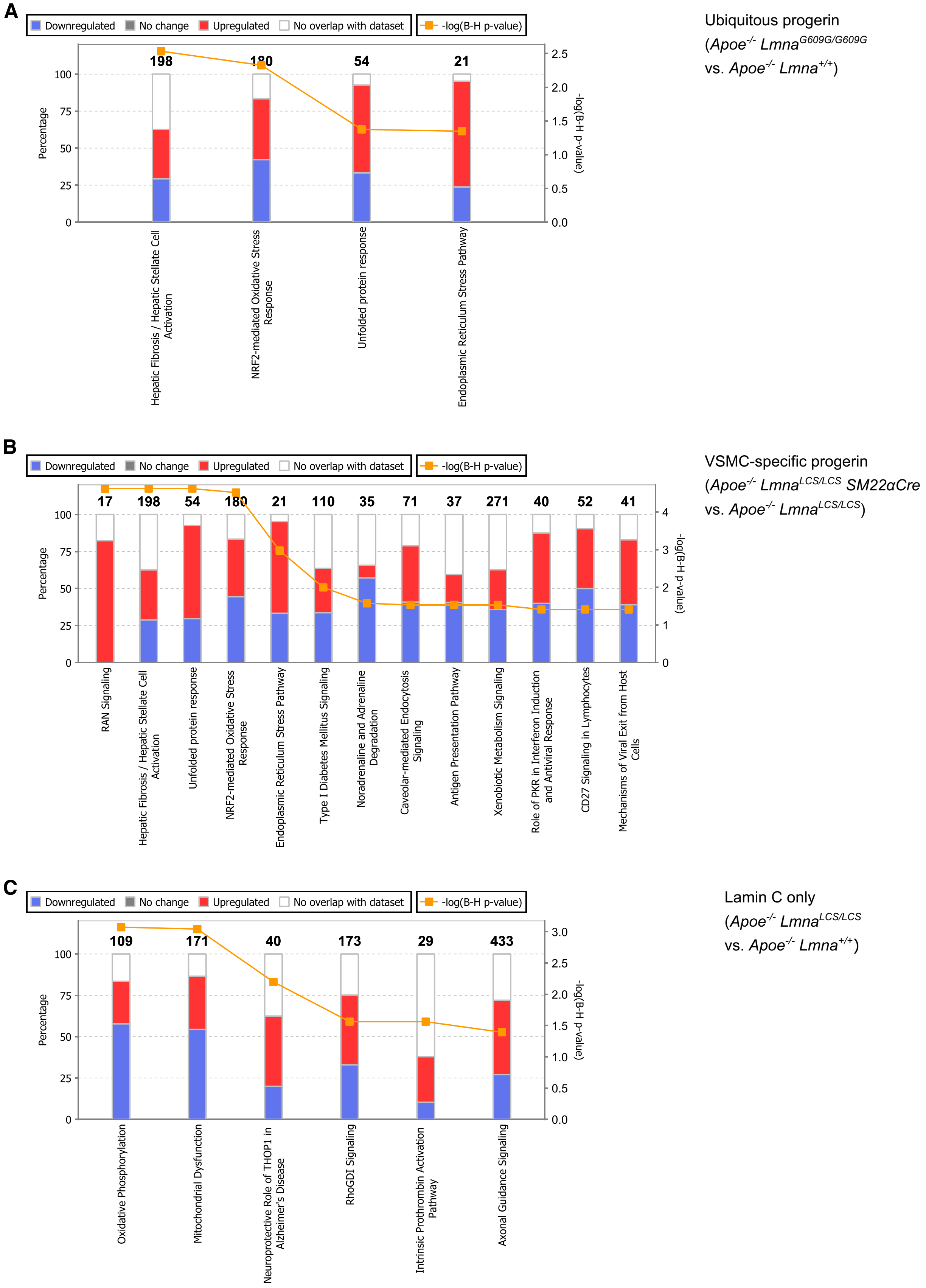
A
Downregulated
No change
Upregulated
No overlap with dataset
-log(B-H p-value)
Percentage
198
180
54
21
Hepatic Fibrosis / Hepatic Stellate Cell Activation
NRF2-mediated Oxidative Stress Response
Unfolded protein response
Endoplasmic Reticulum Stress Pathway
Ubiquitous progerin
($Apoe^{-/-}$ $Lmna^{G609G/G609G}$
vs. $Apoe^{-/-}$ $Lmna^{+/+}$)
B
17
198
54
180
21
110
35
71
37
271
40
52
41
RAN Signaling
Hepatic Fibrosis / Hepatic Stellate Cell Activation
Unfolded protein response
NRF2-mediated Oxidative Stress Response
Endoplasmic Reticulum Stress Pathway
Type I Diabetes Mellitus Signaling
Noradrenaline and Adrenaline Degradation
Caveolar-mediated Endocytosis Signaling
Antigen Presentation Pathway
Xenobiotic Metabolism Signaling
Role of PKR in Interferon Induction and Antiviral Response
CD27 Signaling in Lymphocytes
Mechanisms of Viral Exit from Host Cells
VSMC-specific progerin
($Apoe^{-/-}$ $Lmna^{LCS/LCS}$ SM22αCre
vs. $Apoe^{-/-}$ $Lmna^{LCS/LCS}$)
C
109
171
40
173
29
433
Oxidative Phosphorylation
Mitochondrial Dysfunction
Neuroprotective Role of THOP1 in Alzheimer's Disease
RhoGDI Signaling
Intrinsic Prothrombin Activation Pathway
Axonal Guidance Signaling
Lamin C only
($Apoe^{-/-}$ $Lmna^{LCS/LCS}$
vs. $Apoe^{-/-}$ $Lmna^{+/+}$)

Figure EV1.

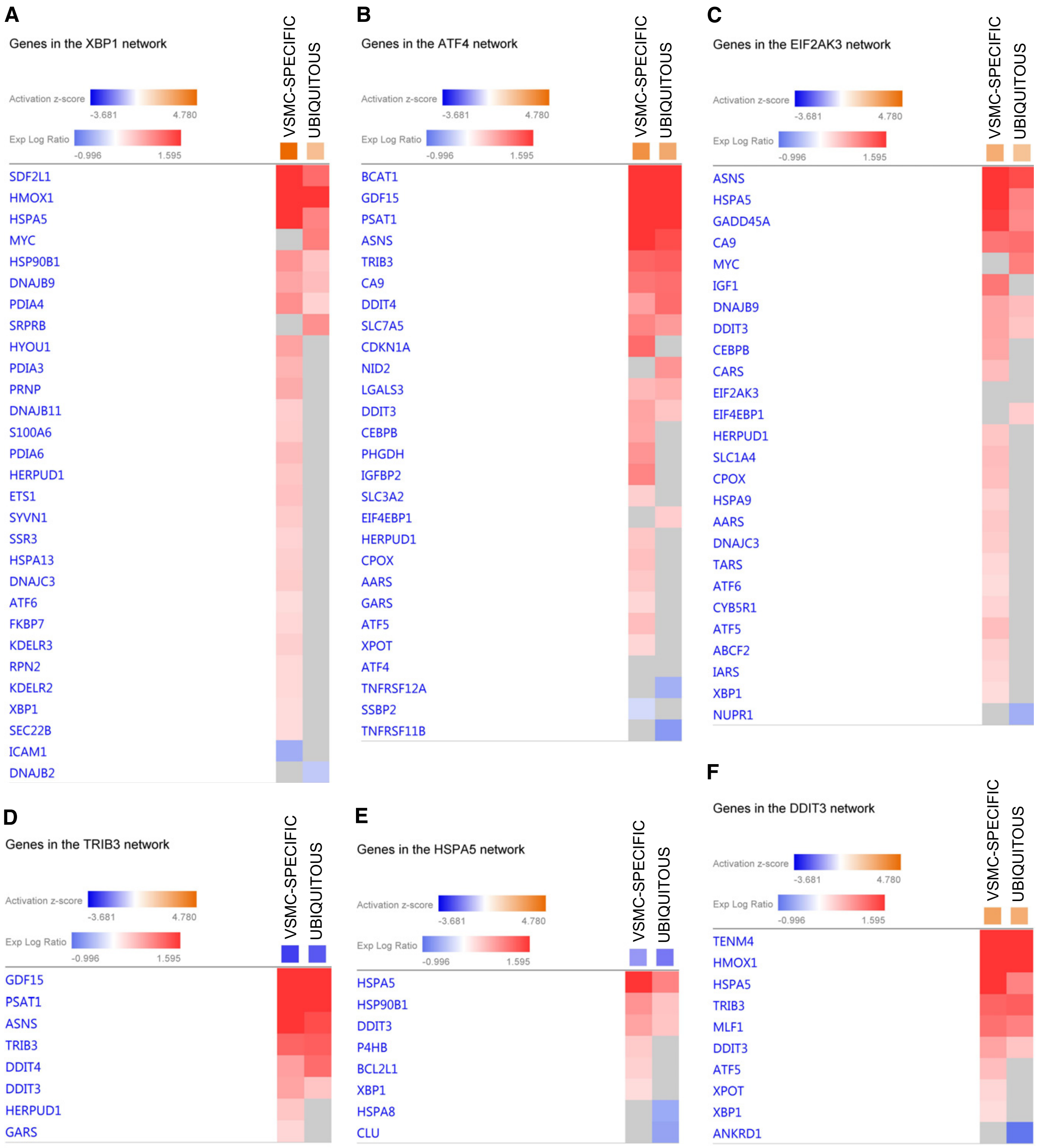

Figure EV2. Genes in the endoplasmic reticulum stress/unfolded protein response-related upstream regulator networks.

A–F Heatmaps (from Ingenuity Pathway Analysis) show expression of genes in (A) X-box-binding protein 1 (gene: *Xbp1*; protein: XBP1), (B) activating transcription factor 4 (gene: *Atf4*; protein: ATF4), (C) eukaryotic translation initiation factor 2-alpha kinase 3 (gene: *Eif2ak3*; protein: EIF2AK3, alternatively protein kinase RNA-like ER kinase, PERK), (D) tribbles pseudokinase 3 (gene: *Trib3*; protein: tribbles homolog 3, TRB3), (E) heat shock protein 5 (gene: *Hspa5*; protein: endoplasmic reticulum chaperone binding-immunoglobulin protein, BiP, alternatively 78 kDa glucose-regulated protein, GRP78), and (F) DNA damage-inducible transcript 3 (gene: *Ddit3*; protein: DNA damage-inducible transcript 3 protein, DDIT3, alternatively C/EBP-homologous protein, CHOP) networks. Gene nomenclature displayed on the figure refers to the human orthologue. VSMC, vascular smooth muscle cell. VSMC-SPECIFIC refers to $Apoe^{-/-}Lmna^{LCS/LCS}SM22\alpha Cre$ vs $Apoe^{-/-}Lmna^{LCS/LCS}$ comparison and UBIQUITOUS refers to $Apoe^{-/-}Lmna^{G609G/G609G}$ vs $Apoe^{-/-}Lmna^{+/+}$ comparison.

 © 2019 The Authors

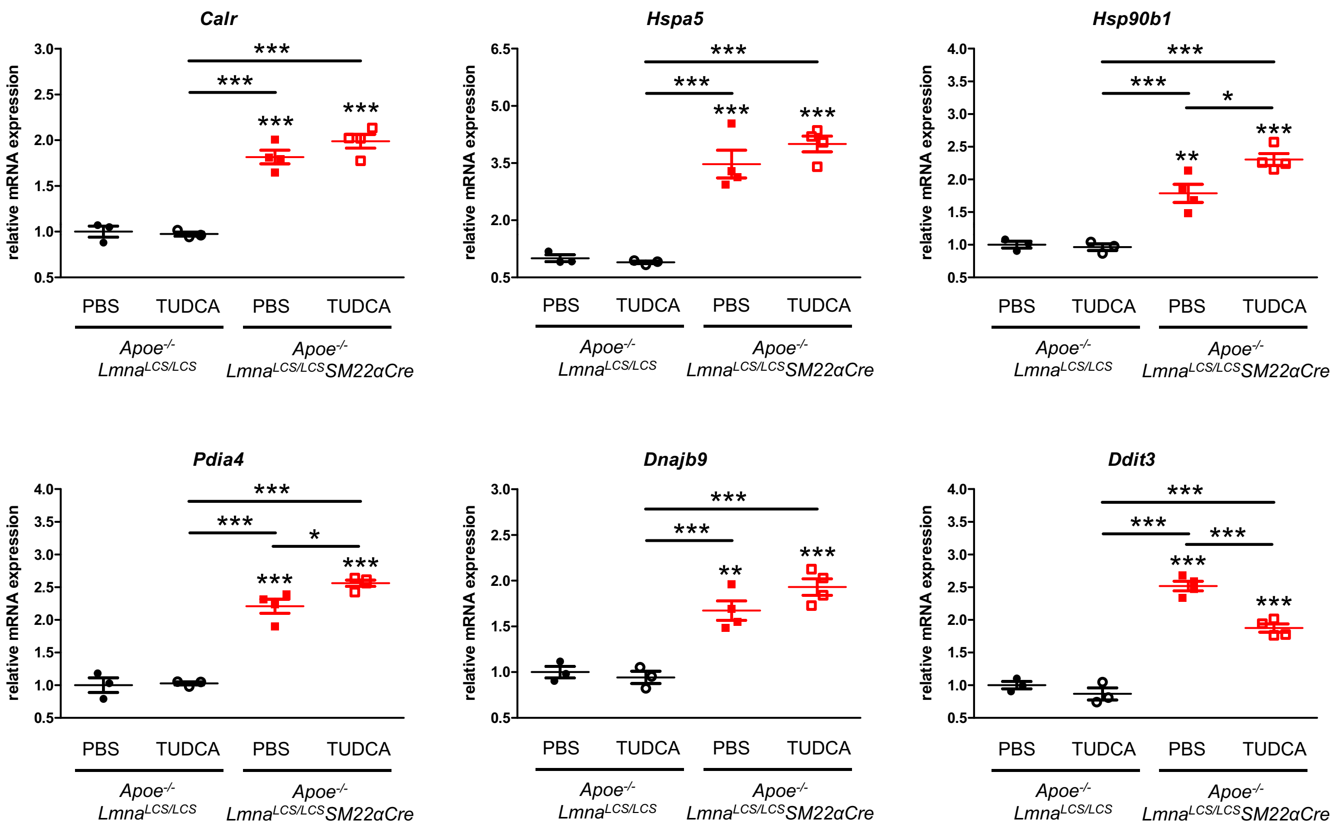

Figure EV3. Treatment with tauroursodeoxycholic acid (TUDCA) reduces *Ddit3* gene expression in medial aortas of *Apoe*$^{-/-}$*Lmna*$^{LCS/LCS}$*SM22αCre* mice.

Eight-week-old *Apoe*$^{-/-}$*Lmna*$^{LCS/LCS}$ and *Apoe*$^{-/-}$*Lmna*$^{LCS/LCS}$*SM22αCre* mice received TUDCA or phosphate-buffered saline (PBS) intraperitoneal injections for 7 consecutive days. Animals were sacrificed at 9 weeks of age, and medial aortas were harvested and pooled (2–3 animals of the same genotype per pool). Six genes related to endoplasmic reticulum stress and the unfolded protein response pathway were analyzed by quantitative real-time PCR. *Hprt* and *Gusb* were used for normalization (n = 3 pooled medial aortas for PBS and TUDCA-treated *Apoe*$^{-/-}$*Lmna*$^{LCS/LCS}$ mice, and n = 4 pooled medial aortas for PBS and TUDCA-treated *Apoe*$^{-/-}$*Lmna*$^{LCS/LCS}$*SM22αCre* mice). Data are mean ± SEM. Statistical differences were analyzed by one-way ANOVA with Tukey's *post hoc* test. *P < 0.05, **P < 0.01, ***P < 0.001.

© 2019 The Authors
