# Supplementary material for: Progerin accelerates atherosclerosis by inducing endoplasmic reticulum stress in vascular smooth muscle cells
Source: EMBO Mol Med. 2019 Mar 12;11(4):e9736. doi: 10.15252/emmm.201809736 (PMC6460349; doi:10.15252/emmm.201809736)
Supplement: Supplementary file 3 — Table EV1 [file EMMM-11-e9736-s003.docx]

**Table EV1 - A gene ontology (GO) cellular compartment overrepresentation test for 240 differentially-expressed genes shared between ubiquitous and vascular smooth muscle cell-specific progeria mouse models.**

| **GO cellular component complete** | **Fold Enrichment** | **P-value** |
| --- | --- | --- |
| sarcoplasmic reticulum (GO:0016529) | 11.50 | 2.20E-02 |
| sarcoplasm (GO:0016528) | 10.04 | 4.66E-02 |
| extracellular matrix (GO:0031012) | 4.36 | 3.10E-04 |
| extracellular organelle (GO:0043230) | 2.27 | 3.72E-05 |
| extracellular exosome (GO:0070062) | 2.24 | 7.82E-05 |
| extracellular vesicle (GO:1903561) | 2.23 | 9.36E-05 |
| extracellular region part (GO:0044421) | 1.99 | 9.41E-06 |
| extracellular space (GO:0005615) | 1.97 | 5.31E-05 |
| extracellular region (GO:0005576) | 1.85 | 8.67E-05 |
| vesicle (GO:0031982) | 1.73 | 2.11E-02 |
| cytoplasm (GO:0005737) | 1.34 | 1.36E-02 |
| membrane-bounded organelle (GO:0043227) | 1.31 | 1.26E-02 |
| organelle (GO:0043226) | 1.31 | 1.93E-03 |
| intracellular (GO:0005622) | 1.26 | 1.24E-02 |
| cell part (GO:0044464) | 1.24 | 4.08E-04 |
| cell (GO:0005623) | 1.24 | 4.19E-04 |
| Unclassified (UNCLASSIFIED) | < 0.2 | 0.00E00 |
