# Supplementary material for: Progerin accelerates atherosclerosis by inducing endoplasmic reticulum stress in vascular smooth muscle cells
Source: EMBO Mol Med. 2019 Mar 12;11(4):e9736. doi: 10.15252/emmm.201809736 (PMC6460349; doi:10.15252/emmm.201809736)
Supplement: Supplementary file 4 — Table EV2 [file EMMM-11-e9736-s004.docx]

**Table EV2 - Treatment with tauroursodeoxycholic acid (TUDCA) reverts features of vulnerable atheroma plaques in *Apoe^-/-^Lmna^G609G/G609G^* and *Apoe^-/-^Lmna^LCS/LCS^SM22αCre* mice.**

|  |  | **Necrotic core**  **(% of the plaque area)** | **SMC content**  **(% of SMA-positive plaque area)** |
| --- | --- | --- | --- |
| ***Apoe^-/-^ Lmna^G609G/G609G^* PBS** | | 31.99±4.32 | 0.89±0.14 |
| ***Apoe^-/-^ Lmna^G609G/G609G^* TUDCA** | | 12.42±3.62 | 1.34±0.19 |
| ***P* value** | | 0.002 | 0.042 |
| ***Apoe^-/-^ Lmna^LCS/LCS^ SM22αCre* PBS** | | 30.79±3.68 | 0.43±0.09 |
| ***Apoe^-/-^ Lmna^LCS/LCS^ SM22αCre* TUDCA** | | 22.90±3.39 | 1.35±0.29 |
| ***P* value** | | 0.072 | 0.016 |

Mice received TUDCA or phosphate buffered saline (PBS) injections 3 times per week starting at 6 (*Apoe^-/-^Lmna^G609G/G609G^*) or 8 (*Apoe^-/-^Lmna^LCS/LCS^SM22αCre* ) weeks of age, and were fed a high-fat diet for 8 weeks starting at 8 weeks of age. At 16 weeks of age, mice were euthanized and aorta was extracted for immunofluorescence studies. Atheroma plaque features were analyzed in three regions of the aortic root and the mean of each mouse was used for further analysis. Necrotic core size was measured in Hoechst 33342-stained sections and smooth muscle cell (SMC) content was quantified in anti-α-smooth muscle actin (SMA)-stained sections (n=6-8 mice for each group).

Data information: Data are mean ± SEM. Statistical differences were evaluated using one-tailed unpaired *t*-test.
