# Supplementary figures and images for: Progerin accelerates atherosclerosis by inducing endoplasmic reticulum stress in vascular smooth muscle cells
Source: EMBO Mol Med. 2019 Mar 12;11(4):e9736. doi: 10.15252/emmm.201809736 (PMC6460349; doi:10.15252/emmm.201809736)

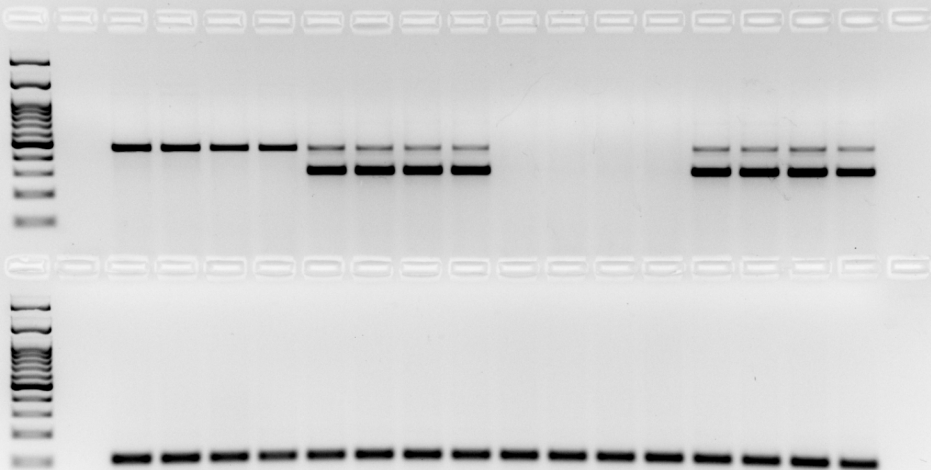

Supplement: Supplementary file 10 — Source Data for Figure 1 [file EMMM-11-e9736-s009.pdf]
