# Supplementary material for: Progerin accelerates atherosclerosis by inducing endoplasmic reticulum stress in vascular smooth muscle cells
Source: EMBO Mol Med. 2019 Mar 12;11(4):e9736. doi: 10.15252/emmm.201809736 (PMC6460349; doi:10.15252/emmm.201809736)
Supplement: Supplementary file 11 — Source Data for Figure 4 [file EMMM-11-e9736-s010.pdf]

Apoe$^{-/-}$ *Lmna*$^{G609G/G609G}$ mice	
Age of death [weeks]	
PBS-treated	TUDCA-treated
13.1	14.3
14.3	14.6
15.9	16.1
16.0	16.3
20.6	17.6
22.6	18.6
23.6	19.0
	19.4

Apoe$^{-/-}$ *Lmna*$^{LCS/LCS}$ *SM22αCre* mice	
Age of death [weeks]	
PBS-treated	TUDCA-treated
24.4	37.9
25.1	47.7
29.9	51.1
37.4	52.7
41.3	53.0
44.4	53.6
44.7	53.7
46.0	61.4
46.9	66.9
47.9	67.3
50.6	67.3
52.0	70.1
52.4	71.1
54.4	79.0
60.1	80.7
60.6	88.9

COMMENTS:

- One *Apoe*$^{-/-}$ *Lmna*$^{LCS/LCS}$ *SM22αCre* animal in the PBS-treated group was excluded from the statistical analysis - it was a significant outlier ($P<0.05$) as assessed by GraphPad outlier calculator. It died at 93.3 weeks of age - far beyond the maximum survival of this mouse model in any of the studies conducted in our laboratory.
- One *Apoe*$^{-/-}$ *Lmna*$^{LCS/LCS}$ *SM22αCre* animal in the TUDCA-treated group was excluded from the study due to health issues associated with injection.
